# Goal Preferences, Affect, Activity Patterns and Health Outcomes in Women With Fibromyalgia

**DOI:** 10.3389/fpsyg.2019.01912

**Published:** 2019-08-21

**Authors:** Maria-Angeles Pastor-Mira, Sofía López-Roig, Fermín Martínez-Zaragoza, Eva León, Ester Abad, Ana Lledó, Cecilia Peñacoba

**Affiliations:** ^1^Department of Behavioral Sciences and Health, Miguel Hernández University, Elche, Spain; ^2^Fibromyalgia Unit, San Vicente del Raspeig Hospital, San Vicente del Raspeig, Spain; ^3^Health Sciences Faculty, Rey Juan Carlos University, Madrid, Spain

**Keywords:** fibromyalgia, goal preferences, activity patterns, affect, health outcomes

## Abstract

Some motivational models understand health behavior as a result of the interaction between goal preferences and mood. However, this perspective has not been explored in fibromyalgia. Furthermore, in chronic pain, it has only been explored with regard to negative affect. Thus, our aims were: (1) to develop a Spanish version of the Goal Pursuit Questionnaire (GPQ); (2) to explore the relationships between goal preferences and health outcomes, testing the moderator role of affect and the mediating role of chronic pain activity patterns. We conducted two cross-sectional studies. In Study 1, after a double translation/back-translation process, we interviewed 94 women attending the Fibromyalgia Unit of the Community of Valencia in order to identify the cultural feasibility and the content validity of the GPQ. Study 2 comprised 260 women. We explored the GPQ structure and performed path analyses to test conditional mediation relationships. Eight activities from the original GPQ were changed while maintaining the conceptual equivalence. Exploratory factor analysis showed two factors: ‘Pain-avoidance goal’ and ‘Mood-management goal’ (37 and 13% of explained variance, respectively). These factors refer to patients’ preference for hedonic goals (pain avoidance or mood-management) over achievement goals. Robust RMSEA fit index of the final models ranged from 0.039 for pain to 0.000 for disability and fibromyalgia impact. Pain avoidance goals and negative affect influenced pain mediated by task-contingent persistence. They also affected disability mediated by task and excessive persistence. Pain avoidance goals and positive affect influenced fibromyalgia impact mediated by activity avoidance. We also found a direct effect of negative and positive affect on health outcomes. Preference for pain avoidance goals was always related to pain, disability and fibromyalgia impact through activity patterns. Affect did not moderate these relationships and showed direct and indirect paths on health outcomes, mainly by increasing persistence and showing positive affect as an asset and not a risk factor. Intervention targets should include flexible reinforcement of achievement goals relative to pain avoidance goals and positive affect in order to promote task-persistence adaptive activity patterns and decreased activity avoidance.

## Introduction

Chronic pain and diseases associated with pain are the most important global causes of disability (Rice et al., 2016). Fibromyalgia is a potentially disabling condition characterized by widespread and diffuse musculoskeletal chronic pain not associated with inflammatory or degenerative changes, alongside other physical, affective and cognitive dysfunctions, such as fatigue, non-refreshed sleep, memory problems, decreased attention, and anxiety and depression (Häuser et al., 2015; Arnold et al., 2016). Patients with this chronic pain condition, the cause of which is unknown, usually show high physical and mental comorbidities, such as headaches, irritable bowel syndrome, and rheumatic diseases or stress (Häuser et al., 2015, 2019). Patients with fibromyalgia often report high functional impact, negative consequences in their daily life and negative effects on mood. In addition, reports suggest a high socio-sanitary burden (Häuser et al., 2015). In Europe, estimates indicate a prevalence of 2.5%, and a high proportion of women (Queiroz, 2013). Despite the growing research and scientific literature of recent years, the diagnosis of fibromyalgia is still controversial (Häuser et al., 2019). The 2016 update of diagnosis criteria, based on a self-reported scale, included: generalized pain as defined by pain occurring in at least four of five body regions; a widespread pain index between 4 and 6 and a symptom severity score of ≥9 or widespread index ≥7 with symptom severity ≥5; finally, symptoms must be present at a similar level for at least 3 months (Wolfe et al., 2016). Currently, fibromyalgia remains an important clinical challenge and the best treatment approach recommended by experts includes graded physical, pharmacological and psychological strategies depending on the severity of the fibromyalgia condition (Macfarlane et al., 2017; Häuser et al., 2019). The main aim is to increase or maintain the physical, psychological and social functions from a rehabilitation perspective.

Emotions have become a significant topic in chronic pain research, in different theoretical models and at different levels of complexity (Dima et al., 2013). One frequent approach has been to explore the emotion contribution to health outcomes and adaptation in patients with this condition. In this sense, there is broad evidence on the direct and indirect effects of positive and negative affect on physical and psychological health. In general, positive affect appears as an asset and part of the resilience mechanisms whereas negative affect is considered as a vulnerability factor for health in different populations (DeSteno et al., 2013; Chan et al., 2016) including in chronic pain and fibromyalgia (Van Middendorp et al., 2008, 2010; Sturgeon and Zautra, 2013; Estévez-López et al., 2015; Hasset et al., 2016). Studies have also been made of contribution of affect to several fibromyalgia symptoms such as cognitive deficits (Galvez-Sánchez et al., 2018) or fatigue (Estévez-López et al., 2019) with similar relationships for positive and negative affect as those mentioned above. Finally, some authors have shown the relationships of affect to chronic pain patients’ activity patterns, exploring its direct effects (Kindermans et al., 2011; Esteve et al., 2017) or its interaction with goal preferences through motivational-affective models (Vlaeyen and Morley, 2004, 2009), enhancing the affective contextual determinants of activity and adaptation.

In fibromyalgia and chronic pain, disability and also adjustment have been explained from a motivational perspective targeting the role of personal valuable goals on these results (Affleck et al., 2001; Hamilton et al., 2005; Crombez et al., 2012; Vlaeyen and Linton, 2012). In this context, activity limitations due to pain are explained by the simultaneity of several competing goals (Crombez et al., 2012) such as the preference for short-term hedonic goals (i.e., pain avoidance) against long-term achievement goals (i.e., to start or to maintain an activity). Karsdorp and Vlaeyen (2011) performed and validated the Goal Pursuit Questionnaire (GPQ) in people with musculoskeletal complaints to identify the individuals’ goal pursuit tendency for hedonic or achievement goals. They explored the relationships of hedonic or achievement goals with pain and disability, and the moderation of negative affect in these relationships. The final version of the GPQ assessed the preference for hedonic goals (pain-avoidance or mood-management goals) in contrast to achievement goals in 16 daily hypothetical situations. They found the endorsement of either pain avoidance or achievement goals were related to pain perception and disability, and that negative affectivity was a significant moderator for pain perception (Karsdorp and Vlaeyen, 2011). As the same authors pointed out, this interaction is similar to the predictions of the mood-as-input model (MAI) which had previously been proposed as a framework to understand the relationships between chronic pain and avoidance or overuse behaviors (Vlaeyen and Morley, 2004; Vlaeyen and Morley, 2009). Indeed Karsdorp and Vlaeyen (2011) designed the GPQ on the basis of this motivational-affective model. The MAI model underlines the informational role of mood in interaction with goals (referred to as stop-rules in the model) in explaining task performance. In people with a preference for hedonic goals, mood informs them whether the activity is pleasurable or not; therefore, positive mood enhances persistence and negative mood encourages disengagement and avoidance. In people with a preference for achievement goals, mood informs them whether goals are reached or not; therefore, positive mood facilitates disengagement and avoidance, and negative mood persistence and overuse. Accordingly, the same mood, in interaction with different goals, encourages either avoidance or persistence behaviors. The model stresses the situational (motivational and affective) determinants of the activity. Affect refers to a predisposition to interpret positively or negatively different stimuli and is more stable than mood. However, we tested the above-mentioned interaction hypotheses, assuming the same effects on avoidance and persistence activity patterns, taking into account that the GPQ was designed to assess people’s habitual goal pursuit.

In a meta-analysis of activity patterns and chronic pain, both activity avoidance and excessive persistence (referred to as overuse or overactivity) were associated with poor health outcomes (Andrews et al., 2012). Moreover, there is broad evidence on the important role of activity patterns in chronic pain outcomes (Kindermans et al., 2011; Esteve et al., 2016, 2017). Regarding fibromyalgia patients, avoidance and persistence behaviors have also been linked to more pain and disability (Van Koulil et al., 2008). To achieve a better understanding of these behaviors and health outcomes in chronic pain, some authors have recommended the investigation of the role of motivational and affective factors from a self-regulation perspective (Vlaeyen and Morley, 2004, 2009; Van Damme and Kindermans, 2015). Research on this issue in fibromyalgia is scarce despite its relevance given the patients’ heterogeneity and high prevalence of both avoidance and persistence activity patterns (Van Koulil et al., 2008), the high disabling impact of the problem, the reported low rates of physical activity (McLoughlin et al., 2011; López-Roig et al., 2016), and the perceived difficulties in performing regular physical activity (Pastor et al., 2015; Peñacoba et al., 2017). Our study tested the Karsdorp and Vlaeyen (2011) affect-goals interaction hypothesis, but in a specific chronic pain sample consisting of women with fibromyalgia. Moreover, as a novel contribution, we added the exploration of the effect of these variables on health outcomes through activity patterns, which was recommended by the same authors (Karsdorp and Vlaeyen, 2011). In addition, we studied not only negative affect but also the role of the positive affect in those relationships. Therefore, in the context of a broader research on self-regulation processes and physical activity in fibromyalgia, we conducted two different studies: (1) to develop a culturally adapted version of the GPQ for a Spanish population of women with fibromyalgia, and (2) to explore the relationships of goal preferences to health outcomes by testing the moderator role of affect and the mediator role of the chronic pain activity patterns.

## Materials and Methods

### Design and Procedure

The two studies are the first part of a broader research project which was approved by the Ethics Committees of the Alicante General Hospital and the Miguel Hernandez University. All participants signed the informed consent.

We conducted a descriptive, observational, cross-sectional design in both studies, with the same inclusion criteria: women, aged between 18 and 70 years and with a fibromyalgia diagnosis from the Fibromyalgia Unit (FU) of the Community of Valencia or from other health services in the case of participants from patients’ associations.

Regarding Study 1, designed to develop a Spanish version of the GPQ (Karsdorp and Vlaeyen, 2011), the authors of the scale sent us the GPQ Dutch original version and authorized its adaptation. We then conducted a double translation/back-translation process and two consensus meetings. Two independent translators provided two target Spanish versions which were translated back to Dutch by two other independent translators. Translators and back-translators translated into their mother tongue. Discrepancies were solved by consensus and we developed a back-translated Dutch version which was compared for equivalence with the original by a bilingual psychologist (López-Roig and Pastor, 2016). Finally, at the FU setting, we performed a field study with four sub-studies: (1) a group structured interview after group self-administration of the GPQ (*n* = 26); (2) a thinking-aloud study (*n* = 16); (3) a group self-administration questionnaire comprising only the activities listed in the GPQ to study their frequency in the daily life of fibromyalgia patients (*n* = 27); and (4) a group self-administration of the Spanish version of the WHYMPI-part III (Pastor et al., 1995), which assessed the frequency of several daily life activities (*n* = 25). With these sub-studies we aimed to assess the feasibility of the GPQ, its clarity (instructions, items and answer scale: sub-studies 1–2) and the appropriateness and content validity of the 16 situations listed in the final version of the original questionnaire (sub-studies 1–4) for the Spanish context and fibromyalgia. In this sense, in the adapted GPQ version for these sub-studies, we also asked participants if they considered each situation as usual or “typical” in people like them and, if not, they were asked to describe another activity with similar emotional or painful consequences. With these added questions in each item of the GPQ pilot version (sub-studies 1–3) and the activities listed in WHYMPI-III (sub-study 4) we aimed to identify common activities in the daily life of these patients to adapt any unknown or unfamiliar situation from the original questionnaire, and to check their conceptual and experiential equivalence (López-Roig and Pastor, 2016). We changed the situation in the original questionnaire if more than 50% of participants had not performed the activity and if more than 50% of the participants considered people in their condition did not perform it.

In Study 2, designed to explore the relationships between goal preferences, affect, activity patterns and health outcomes, questionnaires were self-administered in group sessions to other patients attending the same FU setting (*n* = 163) and in an on-line version for participants from patients’ associations from the Community of Valencia (*n* = 97). Self-administration lasted over 45 min. The total of 260 participants is over the minimum of 200 suggested as sample size for this kind of studies (Izquierdo et al., 2014; Lloret et al., 2014).

## Study 1. Translation and Cultural Adaptation of the GPQ

### Method

#### Participants

Ninety-four women attending the Fibromyalgia Unit (FU) of the Valencian Community participated in the adaptation process of the GPQ. Most were married (65%) and had primary (37.2%) and secondary studies (32%). At the time of the study 23% of women were working. Mean age was 51.3 (*SD* = 10.5) and the mean of perceived pain intensity was 7.3 (*SD* = 1.8); rank 0 = “no pain at all” and 10 = “the worst pain you can imagine.” See description of this measure in Study 2.

#### Variables and Instruments

Socio-demographic and clinical variables were measured with an “*ad hoc*” questionnaire.

##### Goal Pursuit Questionnaire (GPQ)

This instrument measures the habitual goal pursuit of people with pain, taking into account hedonic or achievement goals which can be activated at the same time in one situation. We adapted the final version with 16 items answered on a 6 point Likert scale (1 = strongly disagree, 6 = strongly agree) which has shown adequate psychometric properties (Karsdorp and Vlaeyen, 2011). The questionnaire was designed taking into account the above-mentioned MAI model. Items refer to different daily situations or activities related to work, study or leisure, contrasting hedonic and achievement goals. Items belong to three categories: painful situations (*n* = 8), unpleasant non-painful situations (*n* = 3), and pleasant non-painful situations (*n* = 5). People with pain are required to rate their preference for a hedonic goal or an achievement goal, choosing pain avoidance or mood management (avoiding an unpleasant non-pain situation or maintaining a pleasant non-painful situation). People must imagine ‘as vividly as possible’ the situation presented in a vignette and rate their agreement with a specific thought which refers to their preference for hedonic or achievement goal in this specific situation. The final version of the GPQ showed a structure of two factors, both with 8 items, named: ‘Pain-avoidance goal’ (Factor I; α = 0.88) and ‘Mood-management goal’ (Factor II; α = 0.76). Higher mean scores in each factor indicate stronger preferences for a hedonic goal relative to an achievement goal, that is, to avoid pain (Factor I) or to maintain positive mood (Factor II). Factor I showed low significant and negative relationships with negative affect, sense of responsibility, perfectionism, and fear of negative evaluations. Factor II showed only low significant positive relationships with pain catastrophizing and negative relationships with conscientiousness (Karsdorp and Vlaeyen, 2011). All were coherent with the theoretical predictions.

##### West Haven Yale Multidimensional Pain Inventory (WHYMPI)-part III

The Spanish version contains 16 items and measures the extent of participation in common daily activities of chronic rheumatic patients (Pastor et al., 1995). Items are answered on a numerical rating scale from 0 (never) to 6 (very often). In this study, our interest was limited to the rate for each individual item.

#### Data Analysis

Data were analyzed by SPSS-v24. We conducted a descriptive analysis to analyze sample characteristics and items of the GPQ.

### Results

Instructions and four activities (items 1, 2, 10, 11) were extended to solve problems of comprehension. In both cases, we took into account the comments and the activities expressed in the group structured interview (sub-study 1) and in the thinking-aloud procedure (sub-study 2). For example, regarding instructions, we emphasized the hypothetical condition of the situations (*Remember they are hypothetical situations. It is possible that you have not experienced them or never will. Please, answer imagining yourself in that situation*). In items 1 and 11 we added by hand not only ‘by computer.’ In item 2 we clarified ‘amazing holidays’ by adding *or some amazing thing which has happened to you*, and in item 10, ‘receive an e-mail,’ we added *or WhatsApp*. Finally, eight situations (items 3, 6, 8-10, 12, 14, 15) were changed using the alternative situations proposed by women with fibromyalgia (Table 1). Activities reported by patients as alternatives in items 6, 9, and 14 were also reported as frequent or sometimes in WHYMPI (88, 100, and 48% respectively of participants in sub-study 4).

**TABLE 1:** Original and alternative activities for the GPQ items.

	**Never done**	
**Original situation (item**		**Other people with**
**number) *New situation^a^***	**Participants %**	**fibromyalgia^b^ %**
…paint the windows frame (3)	88.9	85.2
…*clean the windows*		
…load boxes for a move (6)	81.5	70.4
…*load the shopping bags or do the shopping*		
…study for an exam (8)	63.0	51.9
…*read a book*		
…finish the assembly line work (9)	92.6	74.1
…*organize clothes for the washing machine*		
…do a presentation (10)	76.9	53.8
…*do a task*		
…play an instrument in an orchestra (12)	96.3	70.4
… *sewing*		
…repair the car (14)	88.9	77.8
…*clean the car*		
…enjoy writing a report (15)	63.0	51
…*enjoy watching TV*		

*^*a*^In italics new situations reported by patients; final Spanish version is available upon request. ^*b*^Percentage of participants who consider other people with fibromyalgia do not perform it.*

## Study 2. Exploration of the Relationships Between Goal Preferences, Affect, Activity Patterns and Health Outcomes

### Method

#### Participants

A total of 260 women from the FU (*n* = 163) and from patients’ associations of the Valencian Community (*n* = 97) were recruited. Most were married (71.2%) and had primary (36.9%) and secondary studies (38.5%). Mean age was 51.2 (*SD* = 8.7). At the time of the study 31.5% were working and 21% were on sick leave. The mean time from the first symptoms was 15.9 years (*SD* = 11.4) and from the diagnosis it was 7.9 (*SD* = 8.0). The mean of perceived pain intensity was 6.9 (*SD* = 1.4).

#### Variables and Instruments

Socio-demographic and clinical variables were measured with the same *ad hoc* scale as in Study 1. In Study 2, we used the culturally adapted version of the GPQ from Study 1. Regarding validity based on the relation to other constructs, we explored whether high pain catastrophizing would be related to greater endorsement of hedonic goals and whether perfectionism and fear of negative evaluations would be related to greater endorsement of achievement goals (Karsdorp and Vlaeyen, 2011). Our final purpose was to explore whether the preference for hedonic or achievement goals in interaction with positive and negative affectivity would be related to different activity patterns and to health outcomes. Therefore, in the Study 2 we employed:

##### Pain catastrophizing (PCS)

We used the total score of the Spanish adaptation of the Pain Catastrophizing Scale (García-Campayo et al., 2008). This scale contains 13 items answered on a 5-point Likert scale from 0 (not at all) to 4 (all the time) (rank 0–52). Higher scores represent higher catastrophizing (α = 0.95).

##### Perfectionism

We used the total score of the Spanish version of the Frost Multidimensional Perfectionism Scale (FMPS; Gelabert et al., 2011). This scale contains 35 items answered on a 5-point Likert format from 1 (totally disagree) to 5 (totally agree). Higher total score represents higher perfectionism (range 35–175) (α = 0.94).

##### Fear of negative evaluations

Measured with the total score of the Spanish adaptation of the Brief version of the Fear of Negative Evaluation Scale- Straightforward (BFNE-S; Pitarch, 2010). The scale contains 8 items rated on a 5-point Likert scale (1 = not at all characteristic of me; 5 = extremely characteristic of me; range: 8–40). High total score indicates high fear of negative evaluations (α = 0.94).

##### Positive and negative affect

We used the total score of the corresponding trait version subscales (Positive affect: 10 items; Negative affect: 10 items) of the Spanish adaptation of the Positive and Negative Affect Schedule (PANAS; Estévez-López et al., 2016). Items are rated on a 5-point Likert scale from 1 (not at all or very slightly) to 5 (extremely). Scores range from 10-50 in each case. High total score indicates high positive (α = 0.90) or negative affectivity (α = 0.91).

##### Avoidance and persistence activity patterns

We used the Spanish adaptation of the activity patterns scale (Esteve et al., 2016) which contains 24 items rated with a 5-point Likert scale (0 = never, 4 = always) and grouped into eight subscales measuring avoidance (two subscales) persistence (three subscales) and pacing (three subscales). For this study, we only used the subscales related to avoidance and persistence activity patterns: pain avoidance (avoidance behavior related to pain intensity fluctuations; α = 0.75), activity avoidance (avoidance behavior related to the own chronic pain condition; α = 0.55), task-contingent persistence (persistence in finishing task despite pain; α = 0.84), excessive persistence (overuse, persistence without recognition of the own physical limits and with negative rebound effects of this kind of activity; α = 0.65), and pain-contingent persistence (activity is variable depending on pain experience; α = 0.78). Scores on each scale ranged from 0 to 12.

##### Pain intensity

Measured with the mean score of the maximum, minimum, and usual pain intensity during the last week and pain intensity at time of the assessment. These items were answered with an 11-point numerical rating scale (0 = “no pain at all” and 10 = “the worst pain you can imagine”). High mean scores indicate high pain intensity (α = 0.78).

##### Disability

We used the Spanish adaptation of the FIQ-R (Salgueiro et al., 2013). Disability was measured with the sum of the first 9 items divided by 3 (rank 0–30). Items are answered on an 11-point numerical rating scale from 0 to 10. Higher scores represent higher disability (α = 0.89).

##### Fibromyalgia impact

The total score of the above-mentioned questionnaire (rank 0–100). Items are answered on an 11-point numerical rating scale from 0 to 10, with different verbal anchors depending on the item. Higher scores represent higher impact perception (α = 0.93).

#### Data Analysis

We conducted a descriptive analysis for sample characteristics and items of the GPQ. With regard to validity analysis based on internal structure, we performed an Exploratory Factor Analysis (EFA) using the maximum likelihood (ML) method and oblique rotation following the recommended standards (Lloret et al., 2017). Previously, we analyzed whether our data fitted the conditions for linear factor analysis (Lloret et al., 2017) and we tested the floor and ceiling effects of each item (percentage of response above 95% in scores 1 and 6). Factors were selected by the scree plot, Kaiser’ rule and baseline theory. We obtained the Kaiser-Meyer-Olkin index and the Bartlett sphericity test to explore the sampling and data adequacy. Items were retained with loading values greater than 0.45. We also calculated the item-corrected scale correlation with the Pearson coefficient. Pearson correlation was also used for assessing the validity of the GPQ based on the relation to other constructs. Statistical significance was set at *p* < 0.05. Cronbach’s alpha and Omega index was calculated for internal consistency of the scales in our sample. Excepting Omega index, these data were analyzed with the SPSS-v24 (Ventura-León and Caycho-Rodríguez, 2017).

Regarding the interaction effect of affect with goal preferences on activity patterns and the mediation of these on health outcomes, we performed a path analysis. Based on raw data, correlations were converted to a covariance matrix. Model fitting was performed by the lavaan package in R (Rosseel, 2012). The results were reported following the recommendations given in the classic study by Raykov et al. (1991).

MVN package in R (Korkmaz et al., 2014) was used to study assumptions of multivariate and univariate normality. Mardia’s multivariate normality test showed no multivariate normality. Shapiro–Wilk univariate normality tests showed non-normality in all the variables. No missing data were found. Twenty-one outliers were detected by the outliers R package (Komsta, 2011), established on the adjusted quantile method based on Mahalanobis distance, and substituted by the median value.

Conditional mediation models were tested, using two (avoidance activity patterns) or three (persistence activity patterns) mediators depending on the model, and one moderator (positive or negative affect in each case). The modeling process started with a complete model (all the predictors, the moderator, the mediators and one dependent variable) and was improved step by step.

Estimation was calculated by maximum likelihood procedure with robust standard errors and a Satorra–Bentler scaled test statistic, due to the non-normality of the data. Models were improved by removing non-significant parameters and by index modification recommendations, until fit criteria were accomplished, and all parameters were significant. A fit-criteria assessment was conducted according to the Hu and Bentler (1999) study. The goodness-of-fit statistical test assesses the magnitude of unexplained variance; a ratio of χ^2^/gl < 2 suggests an acceptable fit. An RMSEA size below 0.06 suggests a well-fitting model. A CFI above 0.95 indicates a good fit. An SRMR of less than 0.09 also indicates a good fit. The χ^2^ statistic provides a conventional measure of model fit. However, because of its sensitivity to sample size, 2 additional fit indices were used to supplement the χ^2^ statistic. The choice of these 2 indices was based on Hu and Bentler (1999) recommendation of a 2-index presentation strategy, which was found to provide an optimal balance between type I and type II error rates. All these indicators of model fit will be examined later in order to assess whether the model properly represents the data.

**FIGURE 1:**
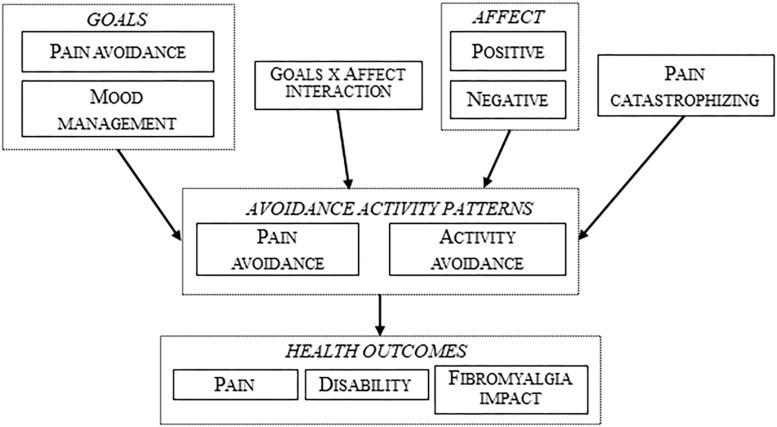
Tested structural model with avoidance activity patterns.

**FIGURE 2:**
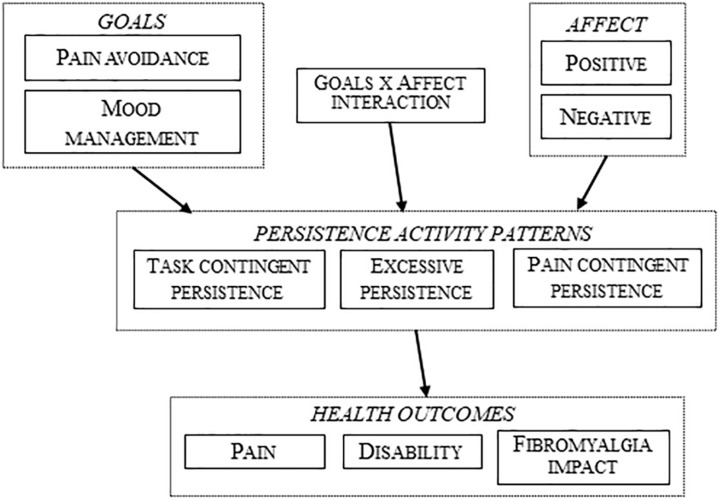
Tested structural model with persistence activity patterns.

Figures 1, 2 represent the tested structural models, with exogenous and endogenous variables. All variables were observed variables and measured on an interval rating scale. The arrows indicate the directionality of the relationships among the variables. In order to simplify the path diagram, the hypothesized effects between each variable are represented with one arrow.

Figure 1 represents the first type of model, a moderated (by positive or negative affect) mediational model with the two avoidance activity patterns as mediational variables (pain avoidance and activity avoidance). These models were tested with positive and negative affect and with three different dependent variables (pain, disability, and fibromyalgia impact). Therefore, 6 analyses were performed.

Figure 2 represents the same scenario but with the three persistence activity patterns (task-contingent persistence, excessive persistence, and pain-contingent persistence) as mediational variables.

Following the premises of the fear-avoidance model, pain catastrophizing was only included in the models with avoidance activity patterns. All the models were improved until all the parameters were significant and the global fit indexes were adequate.

### Results

#### GPQ Analysis

All items had answers on all six response options, and they were normally distributed (Kolmogorov–Smirnoff test). We found no floor or ceiling effects. Item number 4 showed the highest skewness (1.8) and kurtosis (2.5) (Table 2). The KMO test was 0.90 and the Bartlett test was 1869.9 (*p* = 0.000) indicating the adequacy of the sample and the correlation matrix to perform the EFA. The scree plot showed that two mayor factors and one minor factor accounted for 49.9% of the variance. Items 5 (doing calculations; factor loading = 0.42) and 8 (reading a book; factor loading = 0.18) did not reach the minimum established factor loading and were removed from the scale. The third factor was not considered as it only accounted for 2.9% of the variance. No items reached the loading criteria (the highest loading was 0.34 for the item 12) and they had high loadings in the other factors. A second EFA without these two items (KMO = 0.88; Bartlett test = 1706.6; *p* = 0.000) showed two mayor factors accounted for 50% of the variance. Table 2 shows the factor pattern matrix with loadings and descriptive data of the items. Factor I (‘Pain-avoidance goal’: 37% of explained variance; eight items) refers to the choice between pain avoidance goals or achievement goals in different situations. Higher scores reflect stronger preferences for pain-avoidance goals. Factor II (‘Mood-management goal’: 13% of explained variance; six items) refers to the choice between mood-management goals or achievement goals, with higher scores reflecting stronger preferences for mood-management goals. Correlation between both factors was moderate (*r* = 0.42, *p* ≤ 0.01).

**TABLE 2:** Item and factor analysis, descriptive and internal consistency of the GPQ.

**Item**	**I think it is more important…**	**Loading**	***M*^a^**	**SD**	**Sk**	***K***	**r_I–T_**	**h^2^**	**α/Omega**
**Factor II. Pain-avoidance goal**			3.9	1.3	–0.4	–0.8			0.90/0.93
7	…for the pain in my back to be reduced now, than for the house to be cleaned	0.82	4.2	1.8	–0.6	–1.1	0.75	0.66	0.88
3	…for the pain in my shoulder to be reduced now, than the windows to be cleaned	0.82	4.1	1.9	–0.4	–1.3	0.71	0.62	0.88
14	…for the pain in my forearm to be reduced now, than the car to be cleaned	0.81	4.1	1.7	–0.4	–1.2	0.75	0.66	0.88
6	…for the pain in my upper back to be reduced now, than the shopping to be finished	0.78	3.9	1.8	–0.3	–1.4	0.74	0.63	0.88
11	…for the pain in my wrists to be reduced now, than for the album to be completed	0.74	4.2	1.8	–0.6	–1.0	0.72	0.66	0.88
12	…for the pain in my hands to be reduced now, than for the sewing to be finished	0.72	4.3	1.7	–0.6	–0.8	0.72	0.73	0.88
1	…for the pain in my neck to be reduced now, than for my report to be finished on time	0.49	3.5	1.8	0.02	–1.3	0.50	0.33	0.90
16	…for the pain in my elbow to be reduced now, than for the meeting to be arranged	0.48	3.2	1.7	0.2	–1.2	0.56	0.38	0.90
**Factor III. Mood-management goal**			2.5	1.1	0.6	0.1			0.81/0.85
10	…to write a nice message (e-mail or WhatsApp), than to finish the task	0.75	2.4	1.5	0.9	–0.2	0.61	0.50	0.76
4	…to read the exciting book now, than to finish the report on time	0.70	1.8	1.3	1.8	2.5	0.61	0.57	0.77
9	…to decrease my boredom, than to organize clothes for laundry	0.68	2.3	1.6	0.9	–0.3	0.62	0.51	0.76
15	…to enjoy watching the TV program, than to finish my chores	0.62	3.0	1.6	0.3	–1.2	0.55	0.44	0.78
13	…to have interesting conversations now, than to make decisions	0.53	3.3	1.6	0.1	–1.0	0.50	0.34	0.79
2	…to tell my holiday stories or something amazing, than to finish my work	0.49	2.3	1.5	0.9	–0.3	0.50	0.42	0.79

*GPQ, Goal Pursuit Questionnaire; Sk, Skewness; K, Kurtosis; ^*a*^Rank [1–6].*

Descriptive data and correlations are in Table 3. ‘Pain-avoidance goal’ was significant and negatively related to perfectionism, and fear to negative evaluation (both *p* ≤ 0.01). ‘Pain-avoidance goal’ and ‘Mood-management goal’ were significant and negatively related to negative affect (*p* ≤ 0.05 for mood management). ‘Pain-avoidance goal’ was related to more avoidance and less persistence (ranged from *r* = 0.52, *p* ≤ 0.01 for pain avoidance activity pattern to *r* = −0.12, *p* ≤ 0.05 for pain-contingent persistence). ‘Mood-management goal’ factor showed significant correlations with only three activity patterns, ranging from *r* = −0.14, *p* ≤ 0.05 for task-contingent persistence to *r* = −0.15, *p* ≤ 0.05 for pain-contingent persistence (and with the same value but with positive sign for pain avoidance).

**TABLE 3:** Pearson correlation coefficients and descriptive statistics for measured variables in the study.

**Measure**	**1**	**2**	**3**	**4**	**5**	**6**	**7**	**8**	**9**	**10**	**11**	**12**	**13**	**14**	**15**
(1) Pain avoidance goal															
(2) Mood management goal	0.42^∗∗∗^														
(3) Positive affect	0.10	0.03													
(4) Negative affect	–0.24^∗∗∗^	−0.14^∗^	−0.16^∗^												
(5) Pain catastrophizing	–0.11	–0.10	–0.29^∗∗∗^	0.53^∗∗∗^											
(6) Pain avoidance	0.52^∗∗∗^	0.15^∗^	–0.02	–0.17^∗∗^	0.01										
(7) Activity avoidance	0.23^∗∗∗^	0.02	–0.31^∗∗∗^	0.04	0.29^∗∗∗^	0.35^∗∗∗^									
(8) Task-contingent persistence	–0.46^∗∗∗^	−0.14^∗^	0.16^∗^	0.26^∗∗∗^	0.05	–0.60^∗∗∗^	–0.27^∗∗∗^								
(9) Excessive persistence	–0.30^∗∗∗^	–0.09	0.01	0.49^∗∗∗^	0.32^∗∗∗^	–0.35^∗∗∗^	–0.05	0.55^∗∗∗^							
(10) Pain-contingent persistence	−0.12^∗^	−0.15^∗^	0.11	0.31^∗∗∗^	0.17^∗∗^	−0.16^∗^	0.03	0.34^∗∗∗^	0.41^∗∗∗^						
(11) Pain	–0.08	0.08	–0.21^∗∗∗^	0.21^∗∗∗^	0.37^∗∗∗^	0.05	0.17^∗∗^	–0.09	0.16^∗∗^	0.03					
(12) Disability	–0.02	0.00	–0.27^∗∗∗^	0.29^∗∗∗^	0.33^∗∗∗^	–0.04	0.26^∗∗∗^	–0.03	0.24^∗∗∗^	0.02	0.56^∗∗∗^				
(13) Fibromyalgia impact	–0.04	0.00	–0.34^∗∗∗^	0.36^∗∗∗^	0.44^∗∗∗^	0.00	0.37^∗∗∗^	–0.05	0.25^∗∗∗^	0.05	0.63^∗∗∗^	0.88^∗∗∗^			
(14) Perfectionism	–0.26^∗∗∗^	–0.07	–0.04	0.54^∗∗∗^	0.42^∗∗∗^	–0.22	0.11	0.29^∗∗∗^	0.42^∗∗∗^	0.28^∗∗∗^	0.15^∗^	0.29^∗∗∗^	0.33^∗∗∗^		
(15) Fear of negative evaluation	–0.24^∗∗∗^	–0.04	–0.16^∗∗^	0.57^∗∗∗^	0.42^∗∗∗^	−0.14^∗^	0.03	0.17^∗∗^	0.39^∗∗∗^	0.12	0.00	0.19^∗∗^	0.21^∗∗∗^	0.54^∗∗∗^	
Mean	3.94	2.53	23.16	28.83	28.78	6.18	7.38	6.70	6.62	9.00	6.97	19.96	68.88	101.83	21.26
*SD*	1.23	1.06	7.46	9.08	12.18	2.44	2.29	2.63	2.61	2.20	1.41	5.93	16.85	27.55	8.93
Skewness	–0.41	0.62	0.49	0.20	–0.26	0.13	–0.10	–0.30	–0.16	–0.56	–0.44	–0.79	–0.53	0.36	0.32
Kurtosis	–0.78	0.03	–0.23	–0.69	–0.77	0.13	–0.39	–0.34	–0.34	–0.12	0.84	0.34	–0.10	–0.51	–1.00

*^∗∗∗^*p* ≤ 0.001, ^∗∗^*p* ≤ 0.01, ^∗^*p* ≤ 0.05; note: 21 outliers of these data were previously substituted by the median.*

#### Model Fit

The basic starting models were designed according to Figures 1, 2. The fit of the following models was evaluated (Tables 4, 5), and figures were generated by the lavaanPlot package in R (Lishinski, 2018), except for simpler multivariate regression models (models without mediation). Non-standardized parameters can be found in tables and standardized parameters are shown in the figures for greater clarity.

**TABLE 4:** Fitted models with test statistics and path coefficients: goal preferences and affect mediated by avoidance patterns.

**Model and fit**	**Predictor**	**Dependent Variable**	***B***	***SE***	***z***	**Effect size**
**Avoidance patterns**						
Pain with Negative affect	Pain catastrophizing	Pain	0.041	0.007	5.730^∗∗∗^	0.126
χ^2^ = 0.000(0);						
CFI = 1.000; RMSEA = 0.000; SRMR = 0.000						
Disability with Positive affect^a^ Disability with Negative affect	Pain catastrophizing	Disability	0.122	0.029	4.185^∗∗∗^	0.125
χ^2^ = 0.078(1), *p* ≤ 0.780	Pain avoidance goal	Activity avoidance	0.554	0.106	5.255^∗∗∗^	0.157
CFI = 1.000; RMSEA = 0.000; SRMR = 0.005	Pain catastrophizing	Activity avoidance	0.054	0.011	4.812^∗∗∗^	
	Activity avoidance	Disability	0.496	0.157	3.151^∗∗^	
Fibromyalgia impact with Positive affect	Positive affect	Fibromyalgia impact	0.474	0.080	5.888^∗∗∗^	0.251
χ^2^ = 0.116(1), *p* ≤ 0.734	Pain avoidance goal	Activity avoidance	0.554	0.106	5.255^∗∗∗^	0.157
CFI = 1.000; RMSEA = 0.000; SRMR = 0.005	Positive affect	Activity avoidance	0.054	0.011	4.812^∗∗∗^	
	Activity avoidance	Fibromyalgia impact	2.106	0.408	5.156^∗∗∗^	
Fibromyalgia impact with Negative affect	Pain catastrophizing	Fibromyalgia impact	0.314	0.095	3.295^∗∗^	0.290
χ^2^ = 0.509(2), *p* ≤ 0.775	Negative affect	Fibromyalgia impact	0.406	0.126	3.233^∗∗^	
CFI = 1.000; RMSEA = 0.000; SRMR = 0.009	Pain avoidance goal	Activity avoidance	0.554	0.106	5.245^∗∗∗^	0.157
	Pain catastrophizing	Activity avoidance	0.054	0.011	4.811^∗∗∗^	
	Activity avoidance	Fibromyalgia impact	2.231	0.404	5.517^∗∗∗^	

*CFI, Comparative Fit Index; RMSEA, Root Mean Square Error of Approximation; *SE*, Standard Error, SRMR, Standardized Root Mean Square Residual; ^*a*^same model were obtained with positive and negative affect; ^∗∗^*p* ≤ 0.01, ^∗∗∗^*p* ≤ 0.001.*

**TABLE 5:** Fitted models with test statistics and path coefficients: goal preferences and affect mediated by persistence patterns.

**Model and fit**	**Predictor**	**Dependent variable**	***B***	***SE***	***z***	**Effect size**
**Persistence patterns**						
Pain with Positive affect	Positive affect	Task-contingent persistence	0.039	0.020	1.991^∗^	0.014
χ^2^ = 1.369(1), *p* ≤ 0.249	Task-contingent persistence	Pain	–0.074	0.039	−1.894^+^	0.032
CFI = 0.996; RMSEA = 0.039; SRMR = 0.027	Excessive persistence	Pain	0.099	0.040	2.490^∗^	
	Pain	Positive affect	–0.945	0.369	−2.559^∗^	0.037
Pain with Negative affect	Negative affect	Pain	0.035	0.011	3.182^∗∗^	0.219
χ^2^ = 0.336(1), *p* ≤ 0.562	Negative affect	Task-contingent persistence	0.052	0.018	2.912^∗∗^	
CFI = 1.000; RMSEA = 0.000; SRMR = 0.010	Pain avoidance goal	Task-contingent persistence	–0.861	0.121	–7.130^∗∗∗^	
	Task-contingent persistence	Pain	–0.063	0.031	−2.016^∗^	0.053
Disability with Positive affect	Excessive persistence	Disability	0.588	0.139	4.233^∗∗∗^	0.122
χ^2^ = 0.000(0)	Positive affect	Disability	–2.03	0.052	–3.910^∗∗∗^	
CFI = 1.000; RMSEA = 0.000; SRMR = 0.000	Pain avoidance goal	Disability	0.571	0.279	2.046^∗^	
Disability with Negative affect	Negative affect	Disability	0.151	0.046	3.273^∗∗^	0.139
χ^2^ = 0.305(1), *p* ≤ 0.581	Negative affect	Task-contingent persistence	0.052	0.018	2.912^∗∗^	0.219
CFI = 1.000; RMSEA = 0.000; SRMR = 0.007	Pain avoidance goal	Task-contingent persistence	–0.861	0.121	–7.130^∗∗∗^	
	Negative affect	Excessive persistence	0.121	0.013	7.675^∗∗∗^	
	Pain avoidance goal	Excessive persistence	–0.290	0.125	−2.264^∗^	
	Task-contingent persistence	Disability	–0.591	0.145	–4.086^∗∗∗^	
	Excessive persistence	Disability	0.579	0.179	3.229^∗∗^	0.216
Fibromyalgia impact with Positive affect	Positive affect	Fibromyalgia impact	–0.698	0.132	–5.274^∗∗∗^	0.202
χ^2^ = 1.705(2), *p* ≤ 0.426	Pain avoidance goal	Excessive persistence	–0.451	0.132	–3.402^∗∗^	0.045
CFI = 1.000; RMSEA = 0.000; SRMR = 0.023	Pain avoidance goal	Task-contingent persistence	–0.952	0.114	–8.321^∗∗∗^	0.215
	Positive affect	Task-contingent persistence	0.055	0.019	2.972^∗∗^	
	Task-contingent persistence	Fibromyalgia impact	–1.332	0.402	–3.314^∗∗^	
	Excessive persistence	Fibromyalgia impact	2.235	0.407	5.495^∗∗∗^	
Fibromyalgia impact with Negative affect	Negative affect	Fibromyalgia impact	0.654	0.111	5.866^∗∗∗^	0.124
χ^2^ = 0.000(0)						
CFI = 1.000; RMSEA = 0.000; SRMR = 0.000						

*CFI, Comparative Fit Index; RMSEA, Root Mean Square Error of Approximation; *SE*, Standard Error; SRMR, Standardized Root Mean Square Residual; ^+^*p* = 0.058, ^∗^*p* ≤ 0.05, ^∗∗^*p* ≤ 0.01, ^∗∗∗^*p* ≤ 0.001.*

##### Goal models with affect moderation and mediation of the two avoidance patterns

No interaction effects were found between goal preferences (‘Pain-avoidance goal’ or ‘Mood-management goal’) and affect (positive or negative) in any tested model for pain, disability or fibromyalgia impact. Moreover, no multivariate models fitted for pain intensity, either testing the model with positive (no model fitted) or with negative affect (only pain catastrophizing predicted pain in a simple univariate model) (Table 4).

**FIGURE 3:**
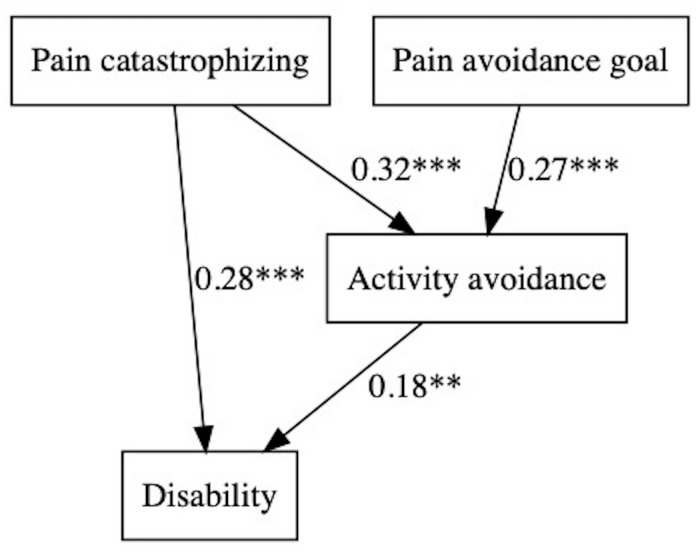
Pain avoidance goal, affect, and activity patterns on disability.

Regarding disability, the models were exactly the same with positive and negative affect: pain catastrophizing predicted disability directly and indirectly, through activity avoidance; in addition, ‘Pain-avoidance goal’ showed a significant and indirect path on disability through activity avoidance (Figure 3). Affect (positive and negative) did not show any significant contribution.

**FIGURE 4:**
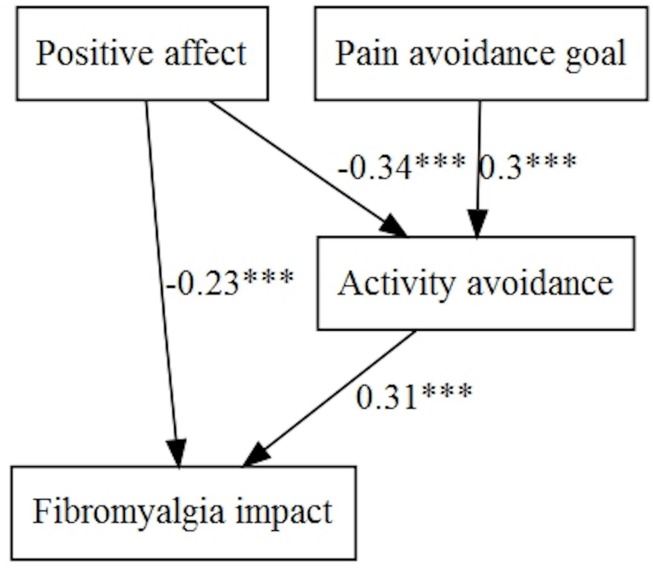
Pain avoidance goal, positive affect, and activity patterns on fibromyalgia impact.

Fibromyalgia impact was predicted by positive affect directly and indirectly through activity avoidance (negatively in both cases). ‘Pain-avoidance goal’ also showed a significant and indirect path with this variable, through activity avoidance (Figure 4).

**FIGURE 5:**
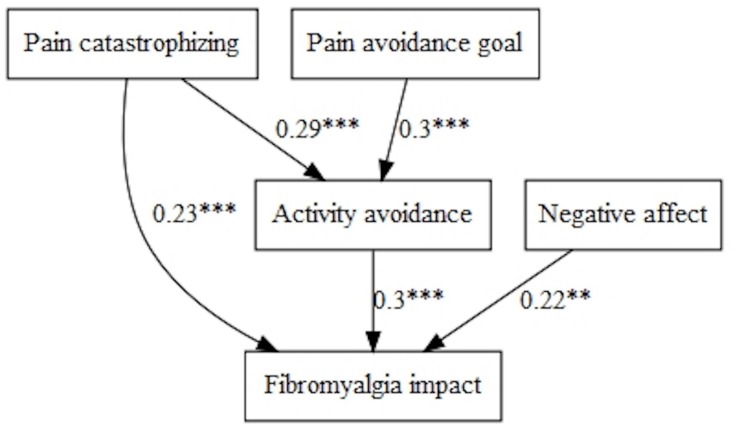
Pain avoidance goal, negative affect, and activity patterns on fibromyalgia impact.

Finally, the fitted model, taking into account the negative affect, showed fibromyalgia impact was influenced directly and indirectly, through activity avoidance, by pain catastrophizing. Moreover, the ‘Pain-avoidance goal’ had an indirect effect on fibromyalgia impact, and negative affect a direct effect (Figure 5).

##### Goal models with affect moderation and mediation of the three persistence patterns

No interaction effects were found between goal preferences (‘Pain-avoidance goal’ or ‘Mood-management goal’) and affect (positive or negative) for pain, disability and fibromyalgia impact when we tested models taking into account the mediation of the persistence activity patterns (Table 5).

**FIGURE 6:**
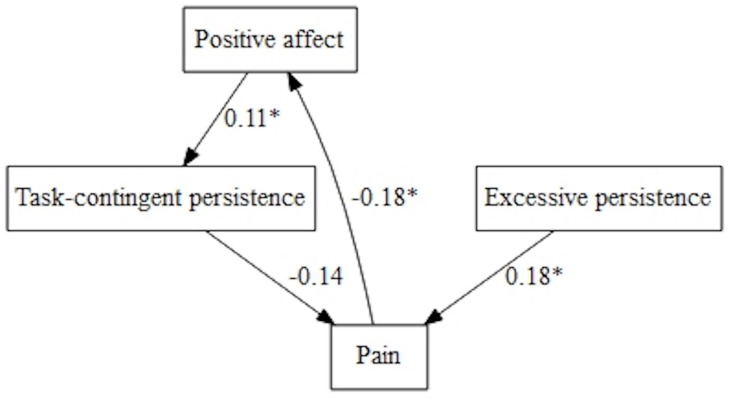
Pain avoidance goal, positive affect, and persistence patterns on pain.

Regarding pain intensity, different models were fitted when we tested goal preference with positive and negative affect. Positive affect showed an indirect path on pain intensity through task-contingent persistence and received the influence of pain intensity with negative sign. In addition, task-contingent and excessive persistence influenced pain with negative and positive sign, respectively. Goal preference did not show any significant contribution in this model. Task-contingent persistence and excessive persistence correlated (*B* = 3.56, *p* < 0.001) (Figure 6).

**FIGURE 7:**
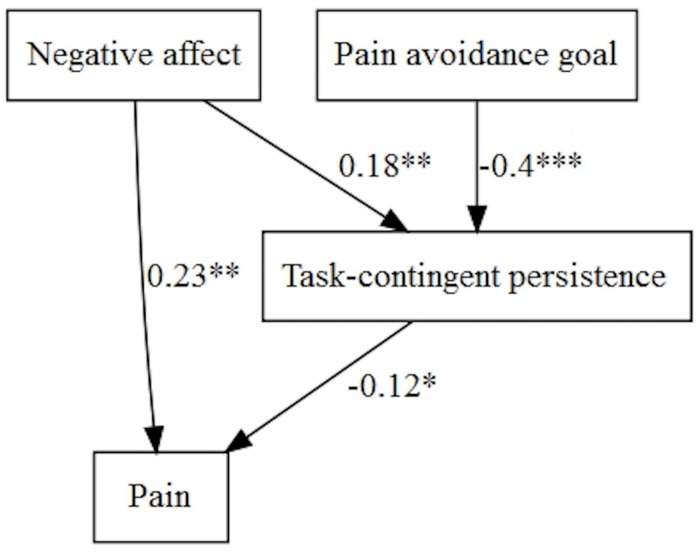
Pain avoidance goal, negative affect, and persistence patterns on pain.

Goal preference played a role in pain intensity when we explored the model with negative affect (Figure 7). Both, negative affect and ‘Pain-avoidance goal’ showed an indirect significant path on pain intensity through task-contingent persistence. Moreover, negative affect showed a positive direct effect on pain.

**FIGURE 8:**
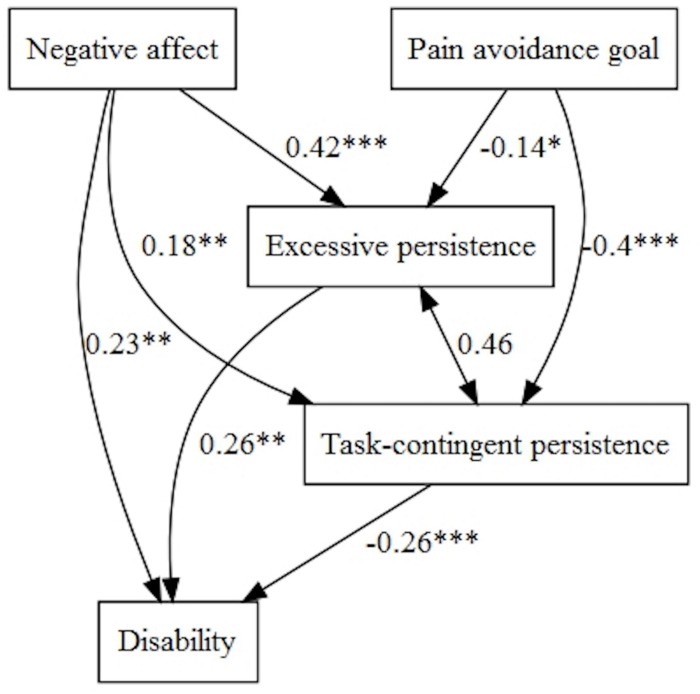
Pain avoidance goal, negative affect, and persistence patterns on disability.

When we tested the models with disability, we only found direct effects of ‘Pain-avoidance goal,’ positive affect and excessive persistence in a simple multivariate model (Table 5). However, when the model was tested with negative affect, this variable (with positive sign) and ‘Pain-avoidance goal’ (with negative sign) influenced disability directly and indirectly, through task-contingent persistence and excessive persistence (Figure 8). Task-contingent persistence and excessive persistence were correlated (*B* = 2.47, *p* < 0.001).

**FIGURE 9:**
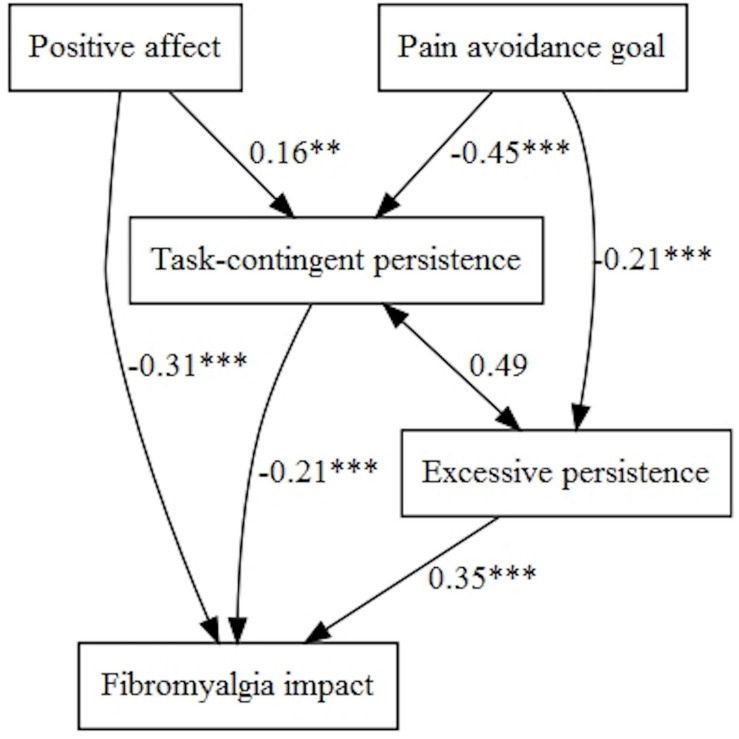
Pain avoidance goal, positive affect, and persistence patterns on fibromyalgia impact.

Fibromyalgia impact was influenced directly by positive affect (with negative sign) and indirectly through task-contingent persistence. Moreover, ‘Pain-avoidance goal’ influenced indirectly through task-contingent persistence and excessive persistence (negatively) fibromyalgia impact (Figure 9).

When we tested the model with negative affect, this was the only variable that predicted fibromyalgia impact, which resulted in a simple univariate model (Table 5).

## Discussion

This research explored the relationships between goal preferences (preference for hedonic goals in contrast with achievement goals), affect (positive and negative), activity patterns (avoidance and persistence) and health outcomes in fibromyalgia. We took into account the predictions from the MAI model, but were aware of the more stable context represented by the GPQ and the affect measures. As a first step toward this aim, we adapted the GPQ to a Spanish population of women with fibromyalgia. The culturally adapted Spanish version resulted in a shorter version with changes in several activities made after the field study conducted with the target population. The main consequence of these changes was more representation of situations related to household tasks than in the original GPQ. Although the original and the final back-translated version did not show a high level of coincidence, we underlined the cultural and experiential equivalence to ensure a comprehensible translation and to maintain the concept while also adapting to the cultural target context (López-Roig and Pastor, 2016). The internal structure of the Spanish version reproduced the original GPQ. The two subscales (‘Pain-avoidance goal’ and ‘Mood-management goal’) showed high reliability and adequate construct validity in our sample. However, we did not obtain significant relationships with pain catastrophizing. As Karsdorp and Vlaeyen (2011) pointed out, the absence of significant relationships confirm they are different constructs and may be an effect of their different conceptualizations. GPQ compares the relative strength of preference for avoidance goals against achievement goals in a motivational context related to different specific situations. On the contrary, catastrophism is measured in a general context, with no motivational context, and without related situations where goals can compete. In addition, we should bear in mind that the total score of catastrophism includes three different dimensions (magnification, rumination and helplessness) and it is possible they do not have the same relationships with goal preferences, and therefore limit the total correlation score. However, this is not an aim of the present study.

In fibromyalgia, the main effects of goal preferences, affect and activity patterns on disability and adjustment, according to relevant psychological models on chronic pain, have been explored in previous research (Vlaeyen and Morley, 2009). However, our study explores these constructs in a more complex framework, taking into account the hypotheses of authors who have applied the MAI model to chronic pain (Vlaeyen and Morley, 2004, 2009; Karsdorp et al., 2010; Karsdorp and Vlaeyen, 2011). As a novel contribution, we have studied the mediation of avoidance and persistence activity patterns in the relationships of the goal preferences and affect with health outcomes. Our results showed no interaction effect of affect and goal preferences on activity patterns. Women with fibromyalgia did not use their positive or negative affect as an informational source for task performance, which supports previous results with mood in experimental studies among people with work-related upper extremity pain (Karsdorp et al., 2010) and among people without pain (Ceulemans et al., 2013; Karsdorp et al., 2013). Similarly, we found activity patterns were explained independently by motivational (preference for pain-avoidance goals) and affective (positive and negative affect) factors. In clinical populations with severe and longstanding pain such as fibromyalgia, mood (affect in this study) can be attributed to pain experience and does not have the informational role hypothesized by the MAI model when mood is attributed to the task. This fact, referred to as ‘the discounting hypothesis,’ suggests that it is possible that there is no interaction between mood and stop-rules (goals) when people attribute their mood to an obvious source (Meeten and Davey, 2011), such as the chronic pain experience in our case.

Results regarding the mediational analyses with the two *avoidance patterns* showed only activity avoidance, in other words avoidance related to the chronic pain condition itself (Kindermans et al., 2011; Esteve et al., 2016), was relevant. In this sense, our findings support the ample evidence available of the fear-avoidance model (Vlaeyen and Linton, 2000, 2012; Leeuw et al., 2007). Catastrophizing thoughts and preference for pain avoidance goals showed a direct and indirect path, increasing activity avoidance, and disability and fibromyalgia impact perception, in line with previous research with chronic musculoskeletal pain and added evidence to the direct link of activity avoidance with disability and fibromyalgia impact perception (Andrews et al., 2012; Esteve et al., 2016, 2017). It is noteworthy that in these models, positive and negative affect did not show any significant path on disability. However, affect played a different role in the general impact of fibromyalgia. Positive affect was related to less activity avoidance and less fibromyalgia impact, and negative affect showed only a direct effect which increased the patients’ perception of fibromyalgia impact.

Finally, no tested model with avoidance patterns was significant for pain intensity. Pain intensity was explained by *persistence patterns*. In the context of a long-term chronic condition (participants had experienced more than 10 years of pain and attended health care tertiary level), the pain is probably integrated in daily experience and persistence would be more relevant as a way of functioning. Affect (positive and negative) and strong achievement goals relative to pain-avoidance goal preferences influenced pain intensity through more endorsement on task-contingent persistence, which was associated with less pain.

Similar to the findings with avoidance patterns, models with persistence were slightly different with positive or negative affect. Only in the model with negative affect, were goal preferences relevant in pain intensity. A strong achievement goal relative to a pain avoidance goal and negative affect increased task-contingent persistence. Negative affect also was directly related to more pain intensity. Regarding disability, the more complex model was obtained with negative affect. This variable and strong endorsement of an achievement goal relative to a pain avoidance goal increased both excessive and task contingent persistence, and these activity patterns were related to more and less disability, respectively. Finally, negative affect also showed a direct path increasing disability. For fibromyalgia impact, the more complex model was obtained with positive affect and, interestingly, showed similar significant paths to negative affect on disability. In these models, task-contingent persistence and excessive persistence predicted better and poorer outcomes respectively in line with previous research (Kindermans et al., 2011; Andrews et al., 2012; Esteve et al., 2016, 2017). These findings provide added evidence of the double adaptive or maladaptive role of persistence on chronic pain outcomes, depending on the kind of persistence and the underlying goals (Van Damme and Kindermans, 2015). In addition, they partially support the avoidance-endurance model of chronic pain (Hasenbring and Verbunt, 2010; Hasenbring and Kindermans, 2018) taking into account the role of negative affect on persistence activity. The role of positive affect as risk factor for overuse, as the model hypothesized, was not supported by our findings. In fact, positive affect appeared as an asset encouraging less avoidance activity and more task-contingent persistence.

Unexpectedly, negative affect increased task-contingent persistence, in contrast with previous research, which found a significant negative relationship between negative affect and this activity pattern (Esteve et al., 2016, 2017). Our result could be explained by the high positive correlation between task-contingent and persistence activity subscales in our sample. In this sense, we must point out that we employed the original factors of the activity patterns scale, developed with heterogeneous musculoskeletal chronic pain patients (Esteve et al., 2016). The above-mentioned significant correlation alongside the low internal consistency of the excessive persistence subscale, may suggest another internal structure of this scale in a unique sample of women with fibromyalgia. The overuse activity pattern characteristic of some groups of patients with fibromyalgia might make the differentiation of the type of persistence for these patients more difficult. However, a positive significant correlation between negative affect and excessive persistence has been previously reported by the same authors, explained as a way of managing affective discomfort involving in excessive activity (Esteve et al., 2016, 2017). This hypothesis could also be true in fibromyalgia, mainly when these patients usually reported high levels of negative emotions and also of persistence (Vlaeyen and Morley, 2004; Hassett et al., 2008; Van Middendorp et al., 2008, 2010). Finally, the direct paths of positive and negative affect with disability and fibromyalgia impact supported previous research on their beneficial and detrimental role respectively in fibromyalgia adaptation (Van Middendorp et al., 2008; Estévez-López et al., 2015, 2017).

This study has some limitations we should bear in mind. First, we conducted a cross-sectional design with correlational data, which does not allow us to establish causal relationships. Second, all measures were self-reported measures. However, the study represents a first view of the motivational and affective determinants of different activity patterns and health outcomes in fibromyalgia, which should be replicated in prospective studies including also objective measures of activity using accelerometers. Third, as we have mentioned, the activity avoidance and excessive persistence subscales of the Activity Patterns Questionnaire (Esteve et al., 2016) showed low internal consistency in our sample. Future studies should perform a replication of the factor structure of this questionnaire in fibromyalgia. Fourth, the sample in the first study was modest; however, in the context of this phase of cultural adaptation of an instrument, a qualitatively representative sample of the target population is essential (Matsumoto and Van De Vijver, 2011). We can find a large variability in sample sizes, for instance, *n* = 5 (Le Gal et al., 2010; Two et al., 2010) or *n* = 14 (Sánchez-Pérez et al., 2017). In addition, we did not check the final translated version with the original authors in order to contrast the right render of the construct; however, we did take into account the participants’ proposals in looking for experiential equivalence in the changed situations of the original questionnaire. Finally, we should point out that as in Karsdorp and Vlaeyen (2011), the effect size of our results was low, possibly due to the complexity of the target.

Despite these limitations, our findings may help to understand motivational and affective issues underlying avoidance and persistence activity in fibromyalgia. In other words, preferences for maintaining a positive mood relative to an achievement goal (‘Mood-management goal’) did not show any role in activity patterns or fibromyalgia health outcomes, in line with the results of Karsdorp and Vlaeyen (2011) with other pain problems. However, strong endorsement of pain avoidance goals relative to achievement goals (‘Pain-avoidance goal’) increased activity avoidance. On the contrary, strong endorsement of achievement goals relative to pain avoidance encouraged both task-contingent persistence and overuse, which showed opposite effects on disability and fibromyalgia impact. Regarding affective issues, positive affect showed significant paths in models with avoidance and persistence patterns. In general terms, positive affect behaved as an asset and a protective factor due to its direct and indirect paths with health outcomes. Women who scored higher on it showed less activity avoidance and more task-contingent persistence and less pain and fibromyalgia impact. Women who scored higher on negative affect showed more task-contingent persistence, which was associated with less pain and disability, but also more excessive persistence or overuse, which was associated with more disability. In addition, negative affect showed direct positive paths to pain and disability, which is also coherent with previous research (Van Middendorp et al., 2008, 2010; Estévez-López et al., 2015, 2017).

Our results did not support the interaction hypothesis of Karsdorp and Vlaeyen (2011). Nevertheless, we belief the ‘Pain-avoidance goal’ subscale can be useful for a self-regulation perspective in fibromyalgia. This scale could be used as a single scale due to its good psychometric properties and its results with avoidance and persistence activity patterns. As has been mentioned, this scale contrasted preferences for pain avoidance goals relative to achievement goals in eight common daily situations for women with fibromyalgia. Therefore, the scale included in the same context two common goals in pain patients: to reduce pain immediately or to persist in the ongoing task despite pain (Hasenbring and Kindermans, 2018). Both goal preferences were relevant in the avoidance and persistence activities of our participants. Therefore, the pain avoidance-achievement goal “bipolarity” of this scale could be useful in applying the self-regulation perspective in chronic pain.

In summary, this study has shown the relevance of pain avoidance and achievement goal preferences in the same context. These preferences always impacted health outcomes through activity patterns, encouraging activity avoidance (when patients endorsed avoid pain relative to achievement goals) and excessive persistence and task-contingent persistence (when patients endorsed achievement goals relative to pain avoidance goals). Positive and negative affect showed direct and indirect effects on health outcomes. Our results supported the mediational role of activity patterns between goal preferences, affect, and health outcomes, and did not support the moderation of affect in these relationships. These findings allow us to improve the understanding of the complex relationships between goal pursuit, vulnerability (catastrophizing and negative affect), psychological assets (positive affect), activity patterns and health outcomes in fibromyalgia. In this sense, reinforcing achievement goals relative to pain avoidance (in a flexible way), and positive affect to promote task-persistence adaptive activity and decreased activity avoidance may prove to be suitable targets in interventions to improve chronic pain adaptation.

## Data Availability

The raw data supporting the conclusions of this manuscript will be made available by the authors, without undue reservation, to any qualified researcher.

## Ethics Statement

The studies involving human participants were reviewed and approved by Ethic committee of Miguel Hernández University and Ethic committe of Alicante General Hospital. The patients/participants provided their written informed consent to participate in this study.

## Author Contributions

M-AP-M, SL-R, and CP developed the design of the research. All authors participated in the Spanish adaptation process of the GPQ. M-AP-M, SL-R, and FM-Z performed the data analyses. M-AP-M, SL-R, and FM-Z wrote the first draft. All authors contributed and reviewed the final draft. All authors approved the manuscript.

## Conflict of Interest Statement

The authors declare that the research was conducted in the absence of any commercial or financial relationships that could be construed as a potential conflict of interest.
